# The Editor and the Advertiser

**Published:** 1900-12

**Authors:** 


### EDITORIAL NOTES AND COMMENTS.

The Business office of THE JOURNAL-RECORD is No. 64 Marietta Street.

The Editorial office is 318-319-320 The Prudential.

Address all Business communications to Dr. M. B. Hutchins, Mgr.

Make remittances payable to THE ATLANTA JOURNAL-RECORD OF MEDICINE.

On matters pertaining to the Editorial and Original Communications address Dr. Bernard Wolff, Atlanta.

Reprints of original articles will be furnished at cost price. Requests for the same should always be made *on the manuscript.*

We will present, post-paid, on request, to each contributor of an original article, twenty (20) marked copies of THE JOURNAL-RECORD containing such article.

### THE EDITOR AND THE ADVERTISER.

MEDICAL journals have been frequently criticized for discrepancies between editorial utterances and advertising pages. The justice of the criticism is rather apparent than real. A little thought will develop the fact that a journal assumes the claims and assertions of its advertising patrons, who seek publicity through its pages, to be correct and established upon fact, but this assumption carries no actual responsibility with it. The interest of the medical journal is concerned with an increase in circulation, based upon the merit or attractiveness of its contents; the interest of the advertiser is parasitic to that of the medical journal. There is no mutual obligation whatever, except that of honesty and fair dealing. The vast number of advertisers in the medical field possess meritorious wares which they desire to have brought to the attention of physicians through legitimate channels, and their composition described frankly, with only such reservation as business principles dictate. Such advertisements pass muster as being within the confines of medical ethics, whatever this may mean.

But there are others so grossly flavored with cheapest quackery that they would clog the alimentary instinct and turn the patent insides of a country newspaper. There can be no apology for an editor who would be instrumental in bringing such offensive stuff beneath the nostrils of his subscribers, or would permit lodgment of it in the neighborhood of the space preempted legitimately by his clean and self-respecting advertising patrons. In our opinion, the latter have a perfectly clear right to protest against such practice, lest they themselves be caught in the overlap of association prejudicial to them.

Of such equivocal material are the correspondence "schools" of osteopathy, magnetic healing and the like, as well as those odds and ends of sure-shot formulas for the cure of drug habits, goitre hernia *et al.*, all ripe for the ash barrel and the garbage cart. These the editor should rigorously exclude as obnoxious to his advertising clients and insults to the intelligence of his subscribers, and either or both may very properly demand it.

